# Intraoperative 5-aminolevulinic acid-induced photodynamic diagnosis of metastatic brain tumors with histopathological analysis

**DOI:** 10.1186/s12957-017-1239-8

**Published:** 2017-09-29

**Authors:** R. Yagi, S. Kawabata, N. Ikeda, N. Nonoguchi, M. Furuse, Y. Katayama, Y. Kajimoto, T. Kuroiwa

**Affiliations:** Department of Neurosurgery, Osaka Medical College, 2-7 Daigaku-machi, Takatsuki, Osaka, 569-8686 Japan

**Keywords:** Metastatic brain tumor, 5-Aminolevulinic acid (5-ALA), Protoporphyrin IX (PpIX), Cell invasion, Adjacent brain tissue, Photodynamic diagnosis (PDD)

## Abstract

**Background:**

Fluorescence-guided surgery using 5-aminolevulinic acid (5-ALA) is a promising real-time navigation method in the surgical resection of malignant gliomas. In order to determine whether this method is applicable to metastatic brain tumors, we evaluated the usefulness of intraoperative fluorescence patterns and histopathological features in patients with metastatic brain tumors.

**Methods:**

We retrospectively reviewed the cases of 16 patients with metastatic brain tumors who underwent intraoperative 5-ALA fluorescence-guided resection. Patients were given 20 mg/kg of 5-ALA orally 2 h prior to the surgery. High-powered excitation illumination and a low-pass filter (420, 450, or 500 nm) were used to visualize the fluorescence of protoporphyrin IX (PpIX), the 5-ALA metabolite. We evaluated the relationships between the fluorescence and histopathological findings in both tumoral and peritumoral brain tissue.

**Results:**

Tumoral PpIX fluorescence was seen in only 5 patients (31%); in the remaining 11 patients (69%), there was no fluorescence in the tumor bulk itself. In 14 patients (86%), vague fluorescence was seen in peritumoral brain tissue, at a thickness of 2–6 mm. The histopathological examination found cancer cell invasion of adjacent brain tissue in 75% of patients (12/16), at a mean ± SD depth of 1.4 ± 1.0 mm (range 0.2–3.4 mm) from the microscopic border of the tumor. There was a moderate correlation between vague fluorescence in adjacent brain tissue and the depth of cancer cell invasion (*P* = 0.004).

**Conclusion:**

Peritumoral fluorescence may be a good intraoperative indicator of tumor extent, preceding more complete microscopic gross total resection.

**Trial registration:**

Institutional Review Board of Osaka Medical College No. 42, registered February 17, 1998, and No. 300, registered April 1, 2008. They were retrospectively registered.

## Background

Intracellular conversion of the porphyrin precursor 5-aminolevulinic acid (5-ALA) to fluorescent protoporphyrin IX (PpIX) generally occurs more efficiently in malignant neoplasms and inflammatory tissue than in normal tissue [1, 2]. Since this unique property of ALA can provide real-time tumor contrast, 5-ALA-induced PpIX fluorescence-guided resection (ALA-FGR) has been widely utilized in the surgery for many types of malignant neoplasms, including malignant glioma [3–5], bladder cancer [6], bronchial cancer, esophageal cancer, and prostate cancer [7]. However, the usefulness of ALA-FGR in the treatment of metastatic brain tumors has not been thoroughly investigated. A major unresolved problem in the surgical treatment of metastatic brain tumors is a local recurrence. Several surgical strategies have been proposed to limit local recurrence. One is en bloc resection using various types of navigation technology; another is the removal of both the tumor and the adjacent brain parenchymal tissue. Recently, it was reported that surgical resection of both the tumor and the adjacent brain parenchymal tissue resulted in a decrease in the rate of local recurrence rate by one third, compared with standard gross total resection (GTR) methods [8]. In this study, we examined intraoperative patterns of fluorescence and related histopathological findings for both the tumor and for the surrounding normal brain tissue. The usefulness and potential applications of fluorescence-guided resection in metastatic brain tumor surgery were then considered based on the results of the study.

## Methods

We retrospectively reviewed a total of 16 consecutive patients who underwent ALA-FGR for metastatic brain tumors at our hospital (Table 1). The use of 5-ALA for intraoperative fluorescence-guided tumor resection was approved by the ethics committee of Osaka Medical College (Institutional Review Board No. 42, registered February 17, 1998, and No. 300, registered April 1, 2008). Each patient received an oral dose of 5-ALA (20 mg/kg body weight) 2 h prior to the induction of anesthesia. During the tumor resection, 5-ALA-induced PpIX fluorescence and autofluorescence were visualized using a commercially available fluorescence-operative microscope (Pentero, Carl Zeiss Inc.; Oberkochen, Germany). In order to more clearly identify cases of vague PpIX fluorescence and autofluorescence, we used additional high-powered illumination excitation sources, including a Xenon light (D-light, Karl Storz GmbH & Co. KG; Tuttlingen, Germany), a high-powered violet laser (BP-300, Ball Semiconductor Ltd.; Frisco, TX, USA), and a high-powered violet LED light (CCS Inc.; Kyoto, Japan). Optimal intraoperative light spectrum settings for each illumination system were greatest in the 405 nm wavelength spectrum, the zone of the most efficient induction of PpIX fluorescence. Fluorescence was observed through a low-pass filter, with blocking wavelengths of 420, 450, or 500 nm. Total microscopic tumor resection was performed with this fluorescence-guided method, combined with a standard navigation system (Stealth Station, Medtronic Inc.; Minneapolis, MN, USA). The visualized fluorescence intensity was classified into three grades as follows: an obvious charcoal red PpIX fluorescence is defined as “strong” fluorescence. A lack of red PpIX fluorescence, even under strong excitation light at an intensity sufficient to visualize autofluorescence, is defined as “non-fluorescence.” A pink or orange fluorescence was defined as “vague” fluorescence. The phenomenon of vague PpIX fluorescence was postulated as deriving from a merging of charcoal red PpIX fluorescence with green autofluorescence, resulting in a change of the fluorescence color spectrum to orange (500 nm low-pass filter) or light red (420 and 450 nm low-pass filters). All PpIX fluorescence samples were examined in the main tumor mass and in the adjacent brain tissue as well. Histopathological analysis of tumors in all patients was performed using hematoxylin and eosin (HE) staining. If present, adjacent brain parenchymal tissue adhering to sections of the removed tumor was evaluated. HE histopathological staining was performed on adjacent brain parenchymal tissue, and the extent of tumor cell invasion within the normal brain was assessed. In addition, the influence of primary-lesion chemotherapy treatment and preoperative brain radiation therapy on the fluorescence of the tumor and the adjacent brain parenchymal tissue was investigated.

**Table 1:** Fluorescence and histopathological features of metastatic brain tumors and adjacent brain in 5-aminorevulinic acid (5-ALA) fluorescence-guided surgery

Case no.	Age sex	Origin of tumor	Histology	Fluorescence of tumor	Fluorescence of adjacent brain tissue	Brain invasion depth (mm)	Preoperative radiation treatment (month)	Preoperative chemotherapy
1	54 F	Breast	Ad	No	Vague	No	−	+
2	70 M	Colon	Ad	No	Vague	Positive (1.7)	−	−
3	63 F	Lung	Ad	Heterogeneous	Vague	Positive (1.2)	−	−
4	56 F	Breast	Ad	No	Vague	Positive (0.7)	−	+
5	76 M	Rectum	Ad	Heterogeneous	Vague	Positive (1.8)	−	+
6	38 M	Unknown	Ad	No	Vague	Positive (2.9)	−	+
7	76 M	Bladder	Sq	No	Vague	No	−	−
8	61 M	Lung	Ad	Heterogeneous	Vague	Positive (3.4)	−	+
9	40 F	Breast	Ad	No	Vague	Positive (0.3)	−	+
10	61 M	Salivary duct	Ad	No	Vague	Positive (0.8)	WBRT (3)	−
11	10 M	Bone	OS	No	No	Positive (0.2)	−	−
12	54 F	Breast	Ad	No	No	No	SRS (27)	+
13	46 M	Lung	Ad	Heterogeneous	Vague	Positive (1.6)	SRS (8)	+
14	67 M	Colon	Ad	No	Vague	Positive (2.0)	SRS (3)	−
15	38 M	Unknown	Ad	No	Vague	Positive (0.3)	−	−
16	49 M	Rectal	Ad	Heterogeneous	Vague	No	−	+

*F* female, *M* male, *Ad* adenocarcinoma, *Sq* squamous cell carcinoma, *OS* osteosarcoma, *WBRT* whole-brain radiation therapy, *SRS* stereotactic radiosurgery

## Results

### Patient population and characteristics

Sixteen consecutive patients undergoing surgery with ALA-FGR for metastatic brain tumors were studied retrospectively. There were 11 men and 5 women, with an average age of 54 years. The origins of the tumors were as follows: 4 breast and alimentary canal, 3 lung, 2 unknown, and 1 bladder, bone, and salivary duct. There were 14 adenocarcinoma, 2 squamous cell carcinoma, and 1 osteosarcoma (Table 1). Prior to the surgical resections for the brain tumors, 9 out of 16 patients had been treated by some anti-neoplastic agents for their original cancers, and 4 out of 16 patients had received radiation therapies. These preoperative treatments did not correlate with positive fluorescence in tumors or adjacent brain tissues.

### PpIX fluorescence in metastatic brain tumors

**Fig. 1:**
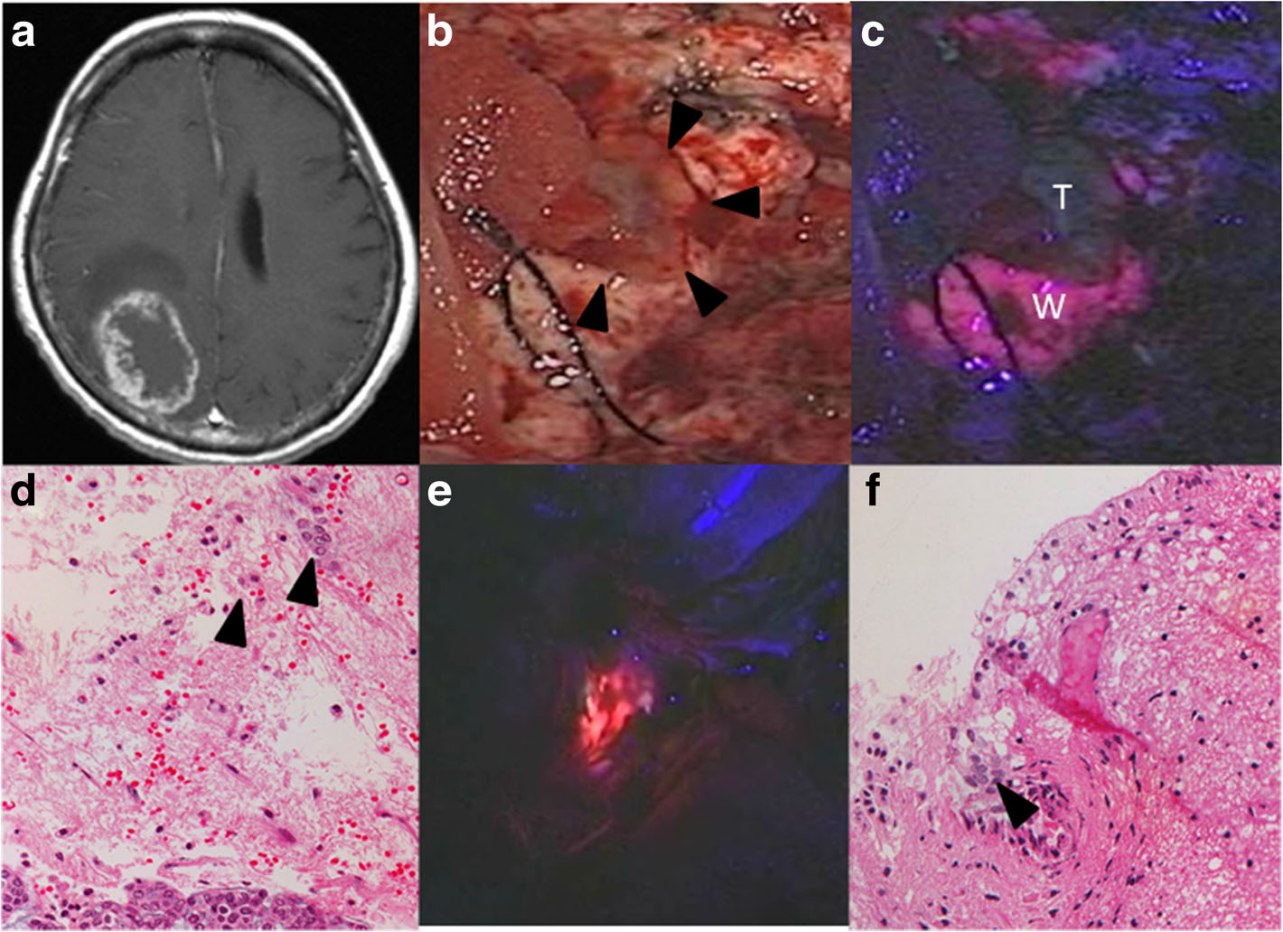
Representative metastatic tumor with absence of PpIX fluorescence (case 4). **a** Gadolinium-enhanced T1-weighted MRI revealing a right occipital metastatic tumor adjusting to the lateral ventricle in a breast cancer patient. **b** This picture shows a tumor removal cavity after the resection of the main tumor bulk which did not show any fluorescence of PpIX. Arrowheads indicate a residual tumor tissue. **c** The residual tumor (T) showed no fluorescence of PpIX as well as its main tumor bulk. In contrast, vague fluorescence was observed in some parts of the adjacent white matters (W) on the surface of the tumor-removal cavity. **d** Histopathology of the PpIX fluorescence-positive white matter (W) in **c**, stained with hematoxylin and eosin (HE). Clusters of cancer cells (arrowheads) were seen in the normal brain parenchyma. **e** Vague PpIX fluorescence was also seen in the exposed ventricular wall after the tumor removal. **f**: Infiltrating cancer cells were seen in the subventricular zone (arrowhead). HE ×200 magnification

**Fig. 2:**
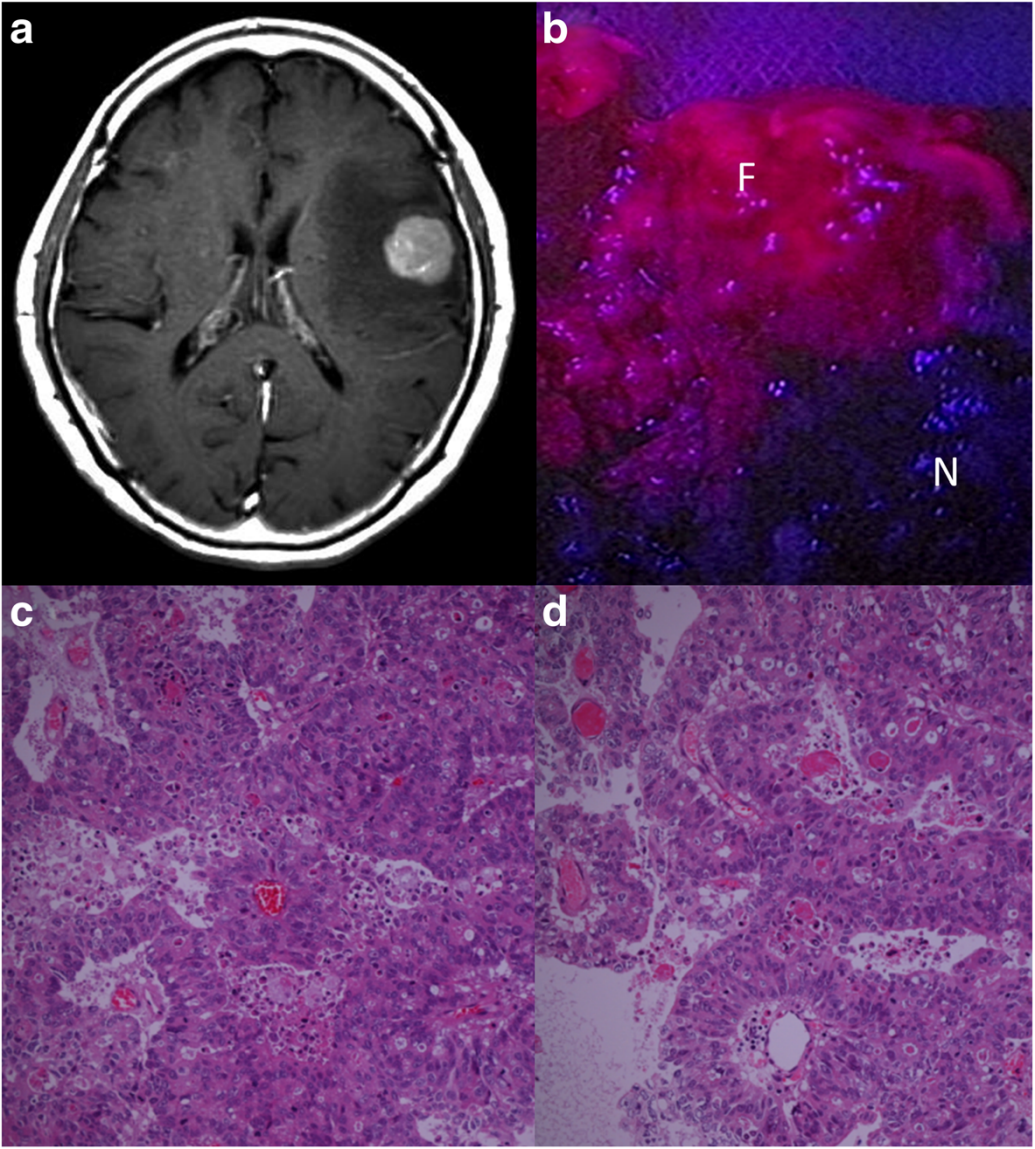
Representative metastatic tumor with strong PpIX fluorescence (case 5). **a** Gadolinium-enhanced T1-weighted MRI showing a left frontal metastatic tumor in a rectal cancer patient. **b** The resected tumor was sectioned and observed under the fluorescence mode. The tumor was divided into fluorescing (F) and nonfluorescing (N) sections. **c** HE staining of the fluorescent section shows typical adenocarcinoma with tubular formation (×200 magnification). **d** HE staining of the nonfluorescing section shows the same histopathological findings as the fluorescent section. There are no necrotic changes (×200 magnification)

In 69% of patients (11/16), there was no visible fluorescence of PpIX in the main tumor mass (Fig. 1b and Table 1). In 31% patients (5/16), tumors, which showed strong fluorescence with a heterogeneous pattern, were observed (Fig. 2b and Table 1). The nonfluorescing areas in these heterogeneous fluorescence cases were identified as mostly necrotic tissue; however, some active tumor areas showed no fluorescence as well (Fig. 2). The PpIX fluorescence sensitivity in metastatic brain tumors was found to be less than 31%. There was no apparent relationship between histopathological type and tumor fluorescence (Table 1).

### PpIX fluorescence in adjacent brain parenchymal tissues

The presence of a vague fluorescence pattern in the brain tissue surrounding the tumor was observed in 88% of patients (14/16) (Table 1). At the deep part in the operation filed, typically vague fluorescence can be difficult to recognize; however, in such situations, vague fluorescence can be distinguished with the aid of additional high-powered excitation light. The vague fluorescent area of the peritumoral lesion ranged from 2 to 6 mm from the tumor. There was no correlation between tumor fluorescence and adjacent brain tissue fluorescence. Vague fluorescence in adjacent brain tissue was observed in 88% of cases (14/16) and was seen even in cases with an absence of central tumoral fluorescence (Table 2).

**Table 2:** Fluorescence patterns between tumor and adjacent brain

	Vague fluorescence in adjacent brain tissue	No fluorescence in adjacent brain tissue	Total
Positive tumor fluorescence	5	0	5
Negative tumor fluorescence	9	2	11
Total	14	2	16

### Tumor cell invasion in the peritumoral brain

**Fig. 3:**
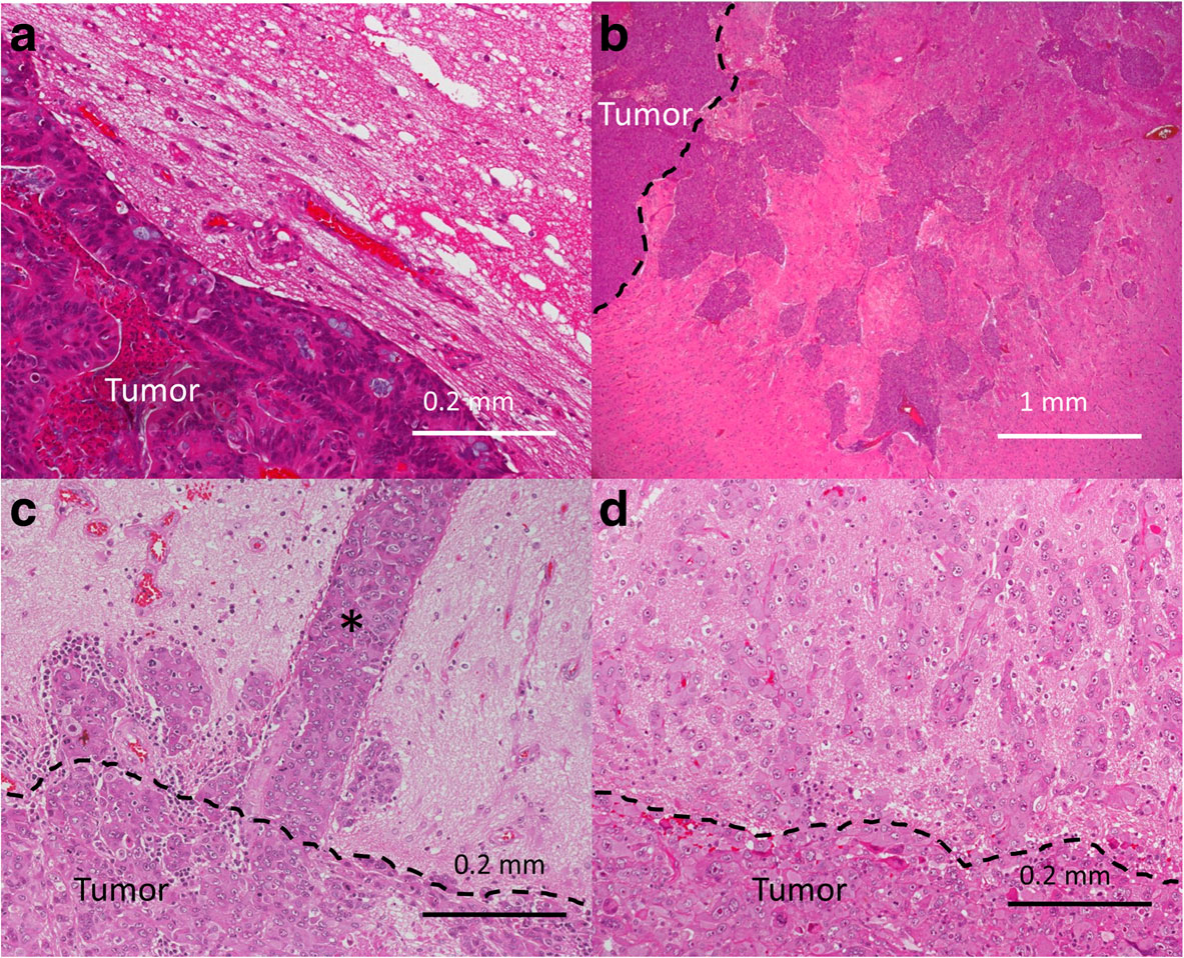
Histopathological findings at the interface between the tumor and peripheral brain. **a** The margins of the metastatic tumor are clear, and no tumor cell invasion can be seen (case 16). **b** The tumor margin is quite unclear, and large clusters of the tumor cells invade deep into the peripheral brain (case 8). The depth of invasion is 3–4 mm. **c** Columnar cell invasion in the microvessels of the peripheral brain can be seen (case 10). The depth of intravascular tumor invasion was approximately 0.82 ± 0.07 mm. The true depth of invasion is unknown because the distal end of invasion was not included in the specimen. **d** The margin of the metastatic tumor is markedly unclear (case 13). Single-cell and small-cell clusters invade the peripheral brain to a depth of 1.6 mm

All of the patients had histopathological sections showing adjacent brain parenchymal tissue adhering to the resected tumor. Cancer cell invasion in the adjacent brain tissue was observed in 75% of patients (12/16). Three patterns of invasion were identified. The first type manifested as direct invasion, extending from the tumor surface to the nearby brain field, with a decrease in cancer cell density being proportional to increasing distance from the tumor surface. A second type was observed in the intravascular spaces (Fig. 3c). The third type was found in the subependymal tissue (Fig. 1f). In this type, isolated cancer cell clusters without apparent continuity with the main tumor mass were detected. However, the presence of contiguous PpIX fluorescence in and around the cancer cells was observed intraoperatively in most cases.

**Fig. 4:**
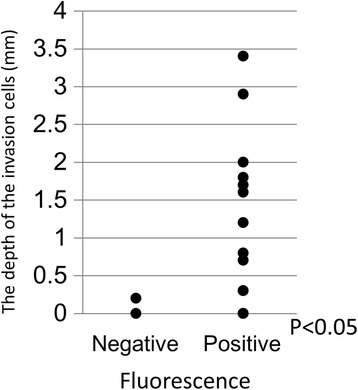
Relationship between vague fluorescence in adjacent brain tissue and depth of cancer cell invasion. There is a correlation between fluorescence in adjacent brain tissue and depth of cancer cell invasion (*P* = 0.004)

The mean ± SD depth of direct invasion was 1.4 ± 1.0 mm, which was less than in peripheral locations (approximately 2–6 mm), where vague fluorescence was typically detected. There was a correlation between the depth of cancer cell invasion and fluorescence of the adjacent brain tissue (*P* = 0.004) (Fig. 4). The depth of cancer cell invasion in the cases of perivascular and subependymal invasion could not be measured.

In 4 patients, there was no histopathological evidence of cancer cell invasion. Despite this finding, in 3 patients (75%), vague peripheral fluorescence was clearly observed. Of the cases of vague fluorescence of the adjacent brain tissue, evidence of pathological tumor cell invasion was detected in 78.6% (11/14).

### The recurrence period and duration of survival

The survival time of 1 patient was less than half a year; for 3 patients (16%), it was between 6 months and 1 year; and for 11 patients (69%), it was more than 1 year. The cause of death of the patient who died within half a year was poor general condition. For the patients who died between half a year and 1 year later, the cause of death for one was a traffic accident, and another one died from exacerbation of the original tumor. The other one died from local recurrence of the tumor and from exacerbation of the original tumor. The survival status of 1 patient is unknown.

## Discussion

### Low positive fluorescence in metastatic brain tumors

Various types of malignant neoplasms can efficiently convert 5-ALA to PpIX, which emits a bright red fluorescence upon irradiation with violet light (405 nm). Because of this, PpIX fluorescence has been found to be useful in identifying the presence of neoplastic cells. The clinical application of this technique has been termed fluorescence-guided surgery or photodynamic diagnosis (PDD) [1]. Target diseases for PDD have included malignant glioma [3–5, 9], meningioma [10], bladder cancer [6], and prostate cancer [7].

The positive rate of PpIX fluorescence in malignant glioma has been found to be greater than 80% [4]. The reason for the high incidence of PpIX fluorescence in malignant neoplasms has been postulated to be a combination of several factors, such as a high uptake of 5-ALA, increased activity of PpIX biosynthesis, and decreased activity of ferrocheratase [1, 11]. However, the predominant causative factor remains unknown.

We found that in metastatic brain tumors, PpIX fluorescence showed only 36% of tumors. The reason for the low positive rate of 5-ALA-induced fluorescence in metastatic brain tumors also remains uncertain. However, it may be due to a fundamental change in the genetic structure of the tumor cells. This supposition is based on the theory that the metastasis of cancer cells is dependent on an inherent change happening in their basic genetic structure. Nguyen et al. distinguished among three classes of metastasis genes: metastasis initiation genes, metastasis progression genes, and metastasis virulence genes [12]. These genetic changes include the gain and loss of function. Therefore, it is likely that one or more of these types of changes has occurred, for example, a decrease in synthesis of PpIX genes or 5-ALA uptake (PEPT1) genes, or an increase in the production of ferrocheratase genes or PpIX transporter (ABCG2) genes. The phenomenon of dedifferentiation may also be relevant for explaining a lack of PpIX fluorescence in metastatic brain tumors. It is known that differentiation of cancer cells can cause an increase in the concentration of PpIX [13–15]. Likewise, the highly undifferentiated nature of metastatic brain tumors may result in a decrease in the concentration of PpIX.

Previously, Kamp et al. reported that 61.5% of metastatic brain tumors exhibited tumor fluorescence. However, interestingly, they observed residual fluorescence of the resection cavity only in 5-ALA-induced fluorescent metastatic tumors. This suggests that they overdiagnosed the adjacent brain fluorescence as tumor fluorescence [16, 17]. Furthermore, a previous report suggests that most metastatic brain tumors (82%) show 5-ALA-induced PpIX fluorescence [18]. This finding, contrary to our observations, may be explained by differences in fluorescence-detection methods. In our study, all samples were from patients undergoing tumor resection performed with a fluorescence-operative microscope and the use of an additional strong excitation light for detecting even faint fluorescence. In contrast, they used a laboratory fluorescent microscope used to view tumor fluorescence in a laboratory setting. Therefore, because our study was performed in an actual clinical intraoperative setting, our result on fluorescence patterns is more accurate.

### PpIX fluorescence in adjacent brain tissue

We found that 88% (14/16) of metastatic brain tumors were surrounded by a vague fluorescent ring of brain tissue in a halo-like formation, while there was no evidence of 5-ALA-induced tumor fluorescence in the main tumor mass in 79% (11/14) of metastatic brain tumors. However, approximately 82% (9/11) of these nonfluorescing metastatic tumors did show peritumoral vague fluorescence. These results indicate that metastatic brain tumors may often have a vague fluorescent rim, but this may be unrelated to the intrinsic fluorescence of the tumor. Vague fluorescence may be due to disruption of the blood–brain barrier (BBB). However, the exact mechanism underlying PpIX fluorescence in the surrounding brain is unknown. Previously, we reported in a clinical observational study that a normal brain with a disrupted BBB shows PpIX fluorescence, even without the presence of a tumor. We have also demonstrated in an in vivo animal experiment that the normal brain parenchymal cells can produce a visible PpIX fluorescence by themselves, if only ALA can enter into the brain parenchyma either by the direct intracerebral injection of ALA or by the BBB disruption using an intra-arterial mannitol injection prior to the ALA administration [2, 19]. On the contrasted T1-weighted image of magnetic resonance imaging (MRI), most of the metastatic brain tumors are visualized with a Gd (gadolinium) enhancer, which means that these tumors have vessels without mature BBB. Thus, 5-ALA also may leak from such tumor vessels and subsequently permeates into the surrounding brain tissue, where it is converted to PpIX by glial cells or infiltrating tumor cells. This suggests that cancer cell invasion has some degree of influence on PpIX fluorescence in adjacent brain tissue. Thus, this relationship could be explained by our hypothesis, whereby invading cancer cells disrupt the BBB in adjacent brain tissue, thereby allowing 5-ALA to enter adjacent brain parenchymal tissue. In the previous studies, it has been reported that the PpIX intensity in a tumor may correlate with a tumor proliferating ability, a vessel density, a primary organ, and a histopathological cancer type. Recently, Wang W et al. reported that glioma stem cells (tumor-initiating cells) showed less PpIX fluorescence compared to non-stem glioma cells [20]. In metastatic brain tumors, the differentiation status of cancer cells might also affect the PpIX production in the tumors.

### 5-ALA fluorescence-guided surgical technique in metastatic brain tumors

We believe that more complete tumor resection of metastatic tumors may be accomplished with the use of 5-ALA fluorescence-guided resection. Our results show that intraoperative findings of either absent or heterogeneously strong PpIX fluorescence with the surrounding brain showing a halo-like vague red fluorescence at depths of 2–6 mm may be highly specific for delineating the extent of typical metastatic brain tumors.

**Fig. 5:**
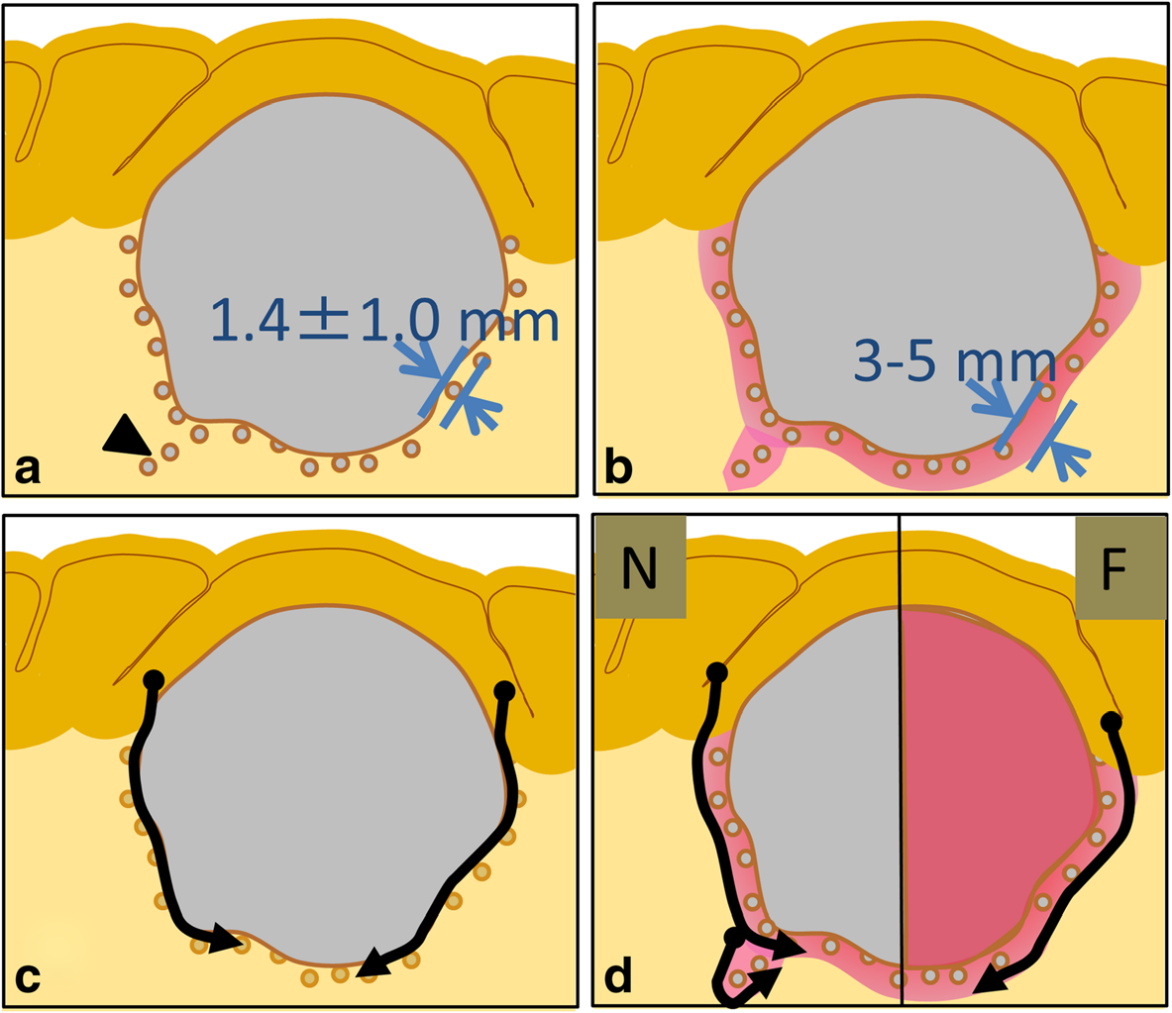
Schematic representation of microscopic GTR using fluorescence guiding. **a** Cancer cell invasion was detected at a mean ± SD depth of 1.4 ± 1.0 mm in 75% of metastatic brain tumor cases. Distant invasion along the vessels and subependymal layers can be observed in some cases (arrow). **b** With the use of 5-ALA, a thin layer of PpIX fluorescence in adjacent brain tissue at a depth of 3–6 mm can be seen. The depth of the PpIX fluorescent layer was greater than the depth of microscopic cancer cell invasion. **c** En bloc tumor resection may reduce the risk of cancer cell invasion in adjacent brain tissue. **d** The thin fluorescent layer in adjacent brain tissue may be a good indicator for achieving microscopic GTR for nonfluorescing (N) and fluorescing (F) sections

So far, despite technical advances in the surgical resection of metastatic brain tumors, prevention of local recurrence continues to be difficult. Hence, a better understanding of the mechanisms of local recurrence could lead to an improved treatment strategy. Three major explanations for local recurrence have been described. The first is incomplete tumor resection. In order to resolve this, more complete GTR has been recommended for the prevention of early local recurrence. Recently, several neuronavigation systems and intraoperative MRI have been applied, in order to improve resection rates, reduce local recurrence, and improve patient survival [21–25]. Image-guided surgery assisted by 5-ALA is a novel technique for identifying residual tumor masses. The second cause of recurrence is the seeding of cancer cells in the operative field. To prevent seeding, en bloc tumor resection, or the no-touch technique, have been recommended. Especially in cases involving the posterior fossa, the incidence of tumor seeding and resultant meningeal carcinomatosis has been found to be much higher than in other regions [26, 27]. The no-touch technique of metastatic brain tumor resection has been reported to be an effective method for preventing tumor cell seeding [28, 29]. Moreover, the no-touch technique or en bloc resection can be further enhanced with the application of fluorescence guiding in peritumoral brain areas (Fig. 5d). Finally, the third factor in local recurrence is cancer cell invasion into the surrounding brain tissue. Although most metastatic brain tumors are macroscopically well demarcated, cancer cells may microscopically invade up to 1 cm into the surrounding brain and along the perivascular space [30, 31].

We recommend 5-ALA fluorescence-guided surgery in metastatic brain tumors. After the incision of the cortex, normal white matter can be distinguished as an autofluorescent area, while adjacent brain parenchymal tissue is identified as an area of vague fluorescence. When incising the thin vague fluorescent layer in the adjacent brain tissue, the surface of the metastatic tumor mass can be distinguished by the presence of either strong fluorescence or a lack of fluorescence (negative fluorescence). We believe that these fluorescence patterns are helpful in avoiding disorientation in tumor dissection by allowing for clear and easy localization. Ultimately, this technique may lead to more complete resection with more effective prevention of local recurrence. Furthermore, in our pathological inspection, we found that 75% of metastatic brain tumors invaded the peritumoral brain at a mean ± SD depth of 1.4 ± 1.0 mm. Based on these findings, resection of adjacent brain tissue to a depth of 3 mm may be sufficient in most cases. Another area of concern is the finding of two types of unexpected distant invasion, intra- or perivascular invasion (Fig. 3c) and subependymal invasion. Typically, both types of invasion are difficult to detect intraoperatively. However, in one case of subependymal invasion, we found that the invaded cluster of cancer cells (Fig. 1f) was surrounded by a rim of vague fluorescent brain tissue (Fig. 1e). Therefore, vague fluorescence of the surrounding brain could become a useful marker for determining the extent of distant cancer cell invasion (Fig. 5b).

### Limitations

Limitations of our study include possible errors in the measurement of the depth of brain invasion. The specimen slice was not always made perpendicular to the tumor surface. Therefore, errors of depth underestimation and overestimation may have occurred. These measurement errors are inevitable with surgical specimens, and a detailed study using tumor samples in autopsy cases or development of a more accurate measurement system would be more desirable. In addition, our pilot study involved a limited number of patients, and studies with a larger patient population and a prospective protocol are needed in the future to more accurately evaluate the effectiveness of this technique.

## Conclusion

In the present case series, we reviewed the intraoperative findings of ALA-FGR for metastatic brain tumors. Positive PpIX fluorescence was more frequently seen in the peripheral brains (14/16) than main tumor mass (5/16), and the existence of PpIX fluorescence in the adjacent brains correlated with the deeper invasion of cancer cells. ALA-FGR might be useful to identify the invading cancer cells and contribute to decrease the tumor relapse after the surgery.

## Abbreviations

5-ALA: 5-Aminolevulinic acid
ALA-FGR: 5-ALA-induced PpIX fluorescence-guided resection
BBB: Blood–brain barrier
GTR: Gross total resection
HE: Hematoxylin and eosin
MRI: Magnetic resonance imaging
PDD: Photodynamic diagnosis
PpIX: Protoporphyrin IX

## References

[1] Peng Q, Warloe T, Berg K, Moan J, Kongshaug M, Giercksky KE, Nesland JM (1997). 5-Aminolevulinic acid-based photodynamic therapy. Clinical research and future challenges. Cancer.

[2] Miyatake S, Kuroiwa T, Kajimoto Y, Miyashita M, Tanaka H, Tsuji M (2007). Fluorescence of non-neoplastic, magnetic resonance imaging-enhancing tissue by 5-aminolevulinic acid: case report. Neurosurgery.

[3] Stummer W, Novotny A, Stepp H, Goetz C, Bise K, Reulen HJ (2000). Fluorescence-guided resection of glioblastoma multiforme by using 5-aminolevulinic acid-induced porphyrins: a prospective study in 52 consecutive patients. J Neurosurg.

[4] Stummer W, Pichlmeier U, Meinel T, Wiestler OD, Zanella F, Reulen HJ (2006). Fluorescence-guided surgery with 5-aminolevulinic acid for resection of malignant glioma: a randomised controlled multicentre phase III trial. Lancet Oncol.

[5] Stummer W, Stocker S, Wagner S, Stepp H, Fritsch C, Goetz C, Goetz AE, Kiefmann R, Reulen HJ (1998). Intraoperative detection of malignant gliomas by 5-aminolevulinic acid-induced porphyrin fluorescence. Neurosurgery.

[6] Kriegmair M, Baumgartner R, Knuchel R, Stepp H, Hofstadter F, Hofstetter A (1996). Detection of early bladder cancer by 5-aminolevulinic acid induced porphyrin fluorescence. J Urol.

[7] Zaak D, Sroka R, Khoder W, Adam C, Tritschler S, Karl A, Reich O, Knuechel R, Baumgartner R, Tilki D (2008). Photodynamic diagnosis of prostate cancer using 5-aminolevulinic acid—first clinical experiences. Urology.

[8] Yoo H, Kim YZ, Nam BH, Shin SH, Yang HS, Lee JS, Zo JI, Lee SH (2009). Reduced local recurrence of a single brain metastasis through microscopic total resection. J Neurosurg.

[9] Pichlmeier U, Bink A, Schackert G, Stummer W (2008). Resection and survival in glioblastoma multiforme: an RTOG recursive partitioning analysis of ALA study patients. Neuro-Oncology.

[10] Kajimoto Y, Kuroiwa T, Miyatake S, Ichioka T, Miyashita M, Tanaka H, Tsuji M (2007). Use of 5-aminolevulinic acid in fluorescence-guided resection of meningioma with high risk of recurrence. Case report J Neurosurg.

[11] Hinnen P, de Rooij FW, van Velthuysen ML, Edixhoven A, van Hillegersberg R, Tilanus HW, Wilson JH, Siersema PD (1998). Biochemical basis of 5-aminolaevulinic acid-induced protoporphyrin IX accumulation: a study in patients with (pre)malignant lesions of the oesophagus. Br J Cancer.

[12] Nguyen DX, Massague J (2007). Genetic determinants of cancer metastasis. Nat Rev Genet.

[13] Anand S, Honari G, Hasan T, Elson P, Maytin EV (2009). Low-dose methotrexate enhances aminolevulinate-based photodynamic therapy in skin carcinoma cells in vitro and in vivo. Clin Cancer Res.

[14] Ortel B, Sharlin D, O'Donnell D, Sinha AK, Maytin EV, Hasan T (2002). Differentiation enhances aminolevulinic acid-dependent photodynamic treatment of LNCaP prostate cancer cells. Br J Cancer.

[15] Sinha AK, Anand S, Ortel BJ, Chang Y, Mai Z, Hasan T, Maytin EV (2006). Methotrexate used in combination with aminolaevulinic acid for photodynamic killing of prostate cancer cells. Br J Cancer.

[16] Kamp MA, Tahsim-Oglou Y, Steiger HJ, Hanggi D (2012). Traumatic brain injuries in the ancient Egypt: insights from the Edwin Smith Papyrus. J Neurol Surg A Cent Eur Neurosurg.

[17] Ferraro N, Barbarite E, Albert TR, Berchmans E, Shah AH, Bregy A, Ivan ME, Brown T, Komotar RJ (2016). The role of 5-aminolevulinic acid in brain tumor surgery: a systematic review. Neurosurg Rev.

[18] Utsuki S, Miyoshi N, Oka H, Miyajima Y, Shimizu S, Suzuki S, Fujii K (2007). Fluorescence-guided resection of metastatic brain tumors using a 5-aminolevulinic acid-induced protoporphyrin IX: pathological study. Brain Tumor Pathol.

[19] Masubuchi T, Kajimoto Y, Kawabata S, Nonoguchi N, Fujishiro T, Miyatake S, Kuroiwa T (2013). Experimental study to understand nonspecific protoporphyrin IX fluorescence in brain tissues near tumors after 5-aminolevulinic acid administration. Photomed Laser Surg.

[20] Wang W, Tabu K, Hagiya Y, Sugiyama Y, Kokubu Y, Murota Y, Ogura SI, Taga T (2017). Enhancement of 5-aminolevulinic acid-based fluorescence detection of side population-defined glioma stem cells by iron chelation. Sci Rep.

[21] Black PM, Johnson MD (2004). Surgical resection for patients with solid brain metastases: current status. J Neuro-Oncol.

[22] Suess O, Kombos T, Kurth R, Suess S, Mularski S, Hammersen S, Brock M (2001). Intracranial image-guided neurosurgery: experience with a new electromagnetic navigation system. Acta Neurochir.

[23] Tan TC, Mc LBP (2003). Image-guided craniotomy for cerebral metastases: techniques and outcomes. Neurosurgery.

[24] Vogelbaum MA, Suh JH (2006). Resectable brain metastases. J Clin Oncol.

[25] Weinberg JS, Lang FF, Sawaya R (2001). Surgical management of brain metastases. Curr Oncol Rep.

[26] Suki D, Abouassi H, Patel AJ, Sawaya R, Weinberg JS, Groves MD (2008). Comparative risk of leptomeningeal disease after resection or stereotactic radiosurgery for solid tumor metastasis to the posterior fossa. J Neurosurg.

[27] van der Ree TC, Dippel DW, Avezaat CJ, Sillevis Smitt PA, Vecht CJ, van den Bent MJ (1999). Leptomeningeal metastasis after surgical resection of brain metastases. J Neurol Neurosurg Psychiatry.

[28] D'Andrea G, Roperto R, Dinia L, Caroli E, Salvati M, Ferrante L (2005). Solitary cerebral metastases from ovarian epithelial carcinoma: 11 cases. Neurosurg Rev.

[29] Salvati M, Cervoni L, Caruso R, Gagliardi FM (1996). Solitary cerebral metastasis from melanoma: value of the ‘en bloc’ resection. Clin Neurol Neurosurg.

[30] Modha A, Shepard SR, Gutin PH (2005). Surgery of brain metastases—is there still a place for it?. J Neuro-Oncol.

[31] Kawaguchi T, Kawaguchi M, Dulski KM, Nicolson GL (1985). Cellular behavior of metastatic B16 melanoma in experimental blood-borne implantation and cerebral invasion. An electron microscopic study. Invasion Metastasis.

